# Giant Choroidal Nevus—A Case Report

**DOI:** 10.3390/reports8020041

**Published:** 2025-03-28

**Authors:** Nina Staneva Stoyanova, Marin Atanassov, Vesela Todorova Mitkova-Hristova, Yordanka Basheva-Kraeva, Maria Kraeva

**Affiliations:** 1 Department of Ophthalmology, Faculty of Medicine, Medical University of Plovdiv, 4000 Plovdiv, Bulgaria; marin_aa@abv.bg (M.A.); vesela_mitkova@abv.bg (V.T.M.-H.); dannybasheva@gmail.com (Y.B.-K.); 2 University Eye Clinic, University Hospital “St. George”, 4000 Plovdiv, Bulgaria; 3 Department of Otorhynolaryngology, Medical Faculty, Medical University of Plovdiv, 6000 Plovdiv, Bulgaria; kraevamaria93@gmail.com

**Keywords:** choroidal nevus, melanoma

## Abstract

**Background and Clinical Significance**: Choroidal nevi are common benign growths originating from pigment cells in the fundus of the eye. They are typically up to 5 mm in diameter, asymptomatic, and incidentally discovered during routine ophthalmological examinations. **Case Presentation**: We present the case of a 48-year-old woman with presbyopic complaints and an incidental finding of a large, pigmented, slightly raised tumor in the fundus of the left eye. Examinations revealed normal visual acuity in both eyes and normal intraocular pressure. Ophthalmoscopy of the left eye identified a large, pigmented lesion measuring 11.55 mm in diameter, with drusen-like deposits along the superior nasal vascular arch. Fluorescein angiography showed atrophic changes in the retinal pigment epithelium without evidence of a pathological vascular network. Ultrasound revealed dimensions of 10.21 mm at the base and 0.57 mm prominence, with no changes observed during a one-year follow-up. Optical coherence tomography (OCT) did not detect any subretinal fluid. The right eye appeared normal. Based on these findings, a giant choroidal nevus in the left eye was suspected, and the clinical approach involved monitoring at 3- to 6-month intervals. **Discussion**: This case highlights the diagnostic challenges associated with a large, pigmented fundus lesion in a relatively young patient. Giant choroidal nevi, defined as lesions larger than 10 mm at the base, may clinically mimic malignant melanoma. However, features such as drusen and atrophic changes in the retinal pigment epithelium suggest a chronic process. Differentiating between giant choroidal nevi and malignant melanoma is essential due to differing therapeutic approaches. Since some choroidal nevi can undergo malignant transformation, close monitoring for signs of malignancy is crucial. **Conclusions**: The size of pigmented nevi is a significant risk factor for malignant transformation, underscoring the importance of long-term follow-up for affected patients.

## 1. Introduction and Clinical Significance

Choroidal nevi are benign tumors originating from the pigment cells of the choroid—melanocytes. They are the most common pigment formations in the fundus and have an incidence of 1.9% to 30% according to various authors [1,2,3]. The first description of choroidal nevi dates back to the 18th century, with early observations made by ophthalmologists studying fundus pigmentation. Over the years, advancements in ophthalmoscopy and histopathology have refined our understanding of these lesions and their clinical significance [3]. According to epidemiological studies, their prevalence is highest in Caucasians (5.6%), compared to African-Americans (0.6%), Hispanics (2.7%), and others (2.1%) [2,3,4,5,6,7]. Their incidence increases with age and is usually detected after 50 years of age, with females being more commonly affected [8,9]. In most cases, choroidal nevi are asymptomatic and are discovered incidentally during a routine eye examination, but they are sometimes associated with reduced vision, visual field defects, development of choroidal neovascular membrane, subretinal fluid, and malignant transformation. Upon examination of the fundus, they appear as pigmented, flat, or slightly raised lesions, with indistinct borders and drusen on the surface. Benign pigmented nevi can be localized in various parts of the fundus: 91% are located in the fundus behind the equator of the eyeball and 9% are found in front of the equator [10]. The distribution of choroidal nevi is relatively equivalent in all quadrants [10]. Their average basic diameter is 5 mm, with a prominence of 1.5 mm [10]. Giant nevi with a diameter of more than 10 mm are observed in about 8% of the cases [11]. Ophthalmological examination methods, such as ophthalmoscopy, ocular ultrasound, fluorescein angiography, and optical coherence tomography, are used to establish the diagnosis, monitor the lesion over time, and detect risk signs for malignancy. In 1% of the cases, there is malignant transformation into malignant melanoma even without the presence of risk factors, and in the presence of such, the rate of malignancy increases. The risk factors for malignant transformation of the choroidal nevi into malignant melanoma are as follows: lesion size—thickness over 2 mm and base over 5 mm; presence of subretinal fluid; subjective complaints such as reduced vision, flashes, floaters, orange lipofuscin pigment, autofluorescence, absence of drusen, and absence of halo during ophthalmoscopy; location less than 3 mm from the optic disc. Although choroidal nevi are usually stationary in nature, the possibility of malignant transformation necessitates strict control of such patients and long-term monitoring.

## 2. Case Presentation

### 2.1. Patient History

A 48-year-old Caucasian woman sought medical attention for reduced near vision, without any other subjective complaints. She denied any concomitant diseases, past trauma, or inflammatory eye diseases.

### 2.2. Clinical Findings

**Figure 1:**
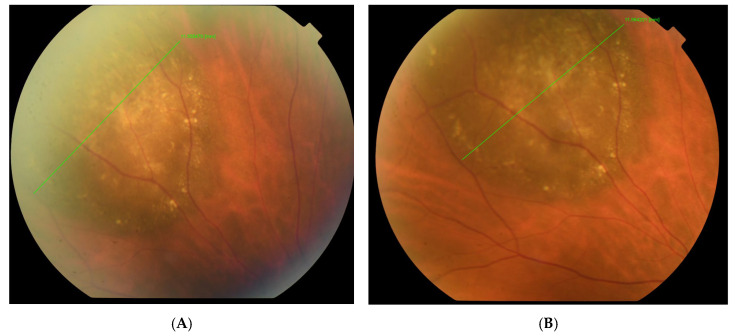
Native photographs of the fundus measuring the diameter of the lesion at the time of diagnosis of the choroidal nevus—11.55 mm, (**A**) and one year later—11.56 mm (**B**).

**Figure 2:**
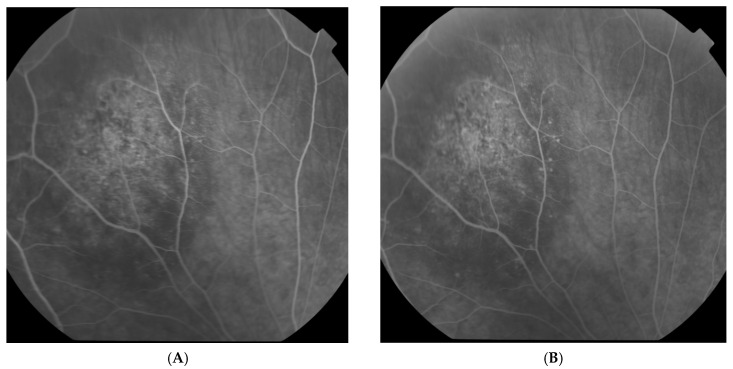
FA—early (**A**) and late (**B**) phases.

**Figure 3:**
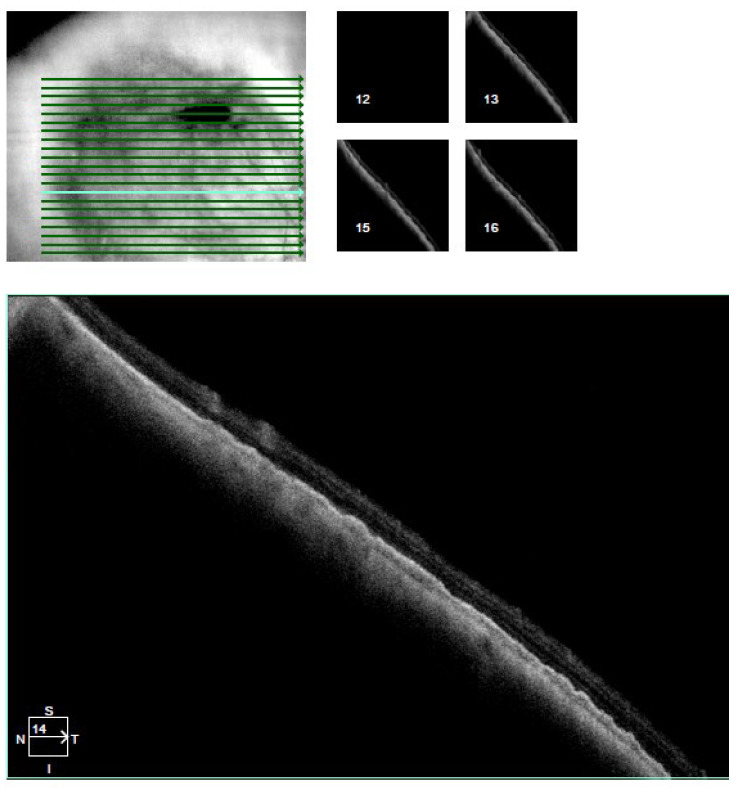
OCT of the choroidal nevus.

**Figure 4:**
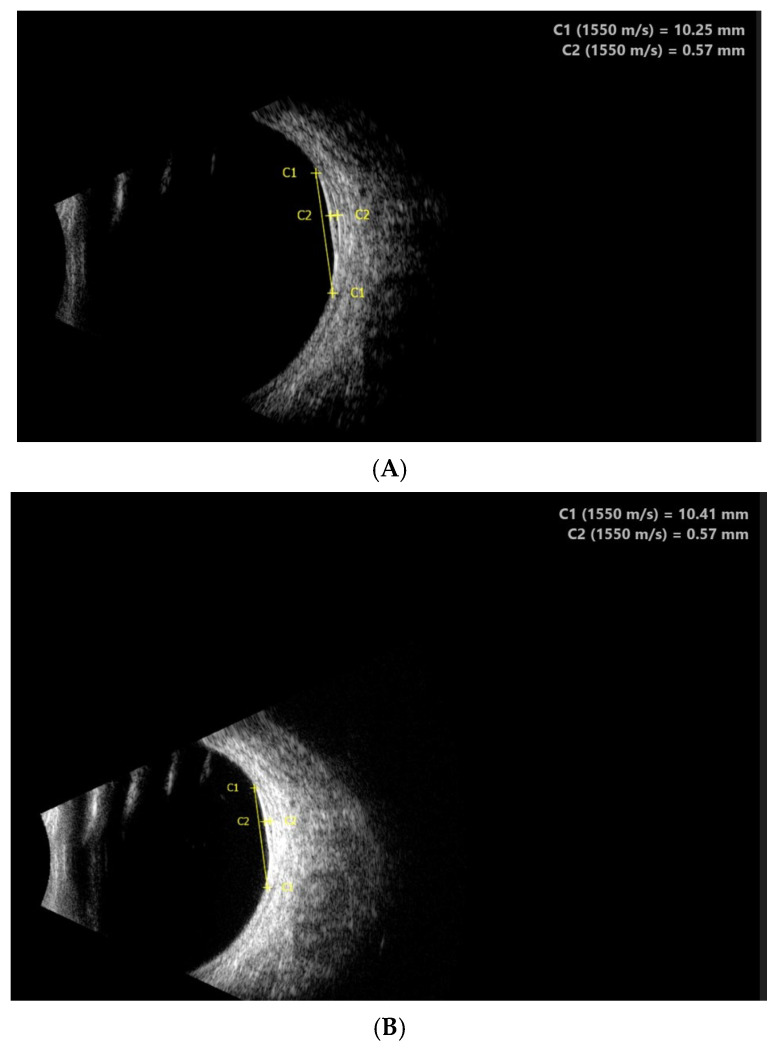
B-ultrasound of the choroidal nevus—at the time of diagnosis (**A**) and after 1 year (**B**).

**Figure 5:**
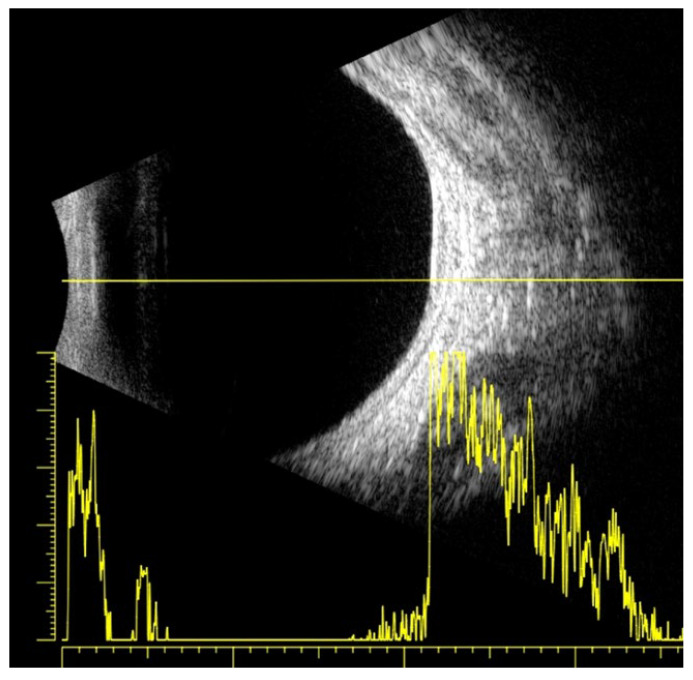
A-mode and B-mode ultrasonography of the choroidal nevus.

The visual acuity of both eyes was 1.0, and the intraocular pressure measured by Goldmann tonometry was 16 mmHg for the right eye and 15 mmHg for the left eye. Biomicroscopy revealed a normal anterior segment of both eyes. Upon ophthalmoscopy, no pathological changes in the right eye were detected, while in the left eye, a normal optic disc and a normal macula with a clear foveal reflex was established. Along the course of the superior nasal vascular arch, a large pigmented lesion of aspid-like color, with relatively defined borders, slightly prominent, and with the presence of drusen-like deposits on its surface, was found. A high-resolution fundus photograph was obtained, and the diameter of the pigmented lesion was measured—11.55 mm (Figure 1A). Fluorescein angiography (FA) (TOPCON TRC-50 DX, Capelle aan den IJssel, The Netherlands) revealed atrophic changes in the retinal pigment epithelium (RPE) without any evidence of the formation having its own vascular network (Figure 2). OCT (CIRRUS 6000, ZEISS, Jena, Germany) did not detect subretinal fluid or choroidal neovascular membrane (Figure 3). B-ultrasound (ABSolu, Quantel Medical, CMI, Cournon-d’Auvergne, France) revealed a flat elevated hyperechoic lesion in the superior nasal quadrant, measuring 10.21 mm at the base, with a prominence of 0.57 mm and not leading to any sign of choroidal excavation (Figure 4). A-mode ultrasound showed a high reflectivity and kappa angle = 0 (Figure 5). During the first year, the patient was monitored every 3 months, with all examinations showing a stable lesion. One year after the initial diagnosis, the size of the nevus was 11.56 mm, measured on native photographs, and its prominence was 0.57 mm, determined by ultrasound.

### 2.3. Diagnostic Assessment

The diagnosis of choroidal pigmented nevus was established based on the clinical finding and the examinations performed, as well as the lack of dynamics of the lesion for over a year.

### 2.4. Therapeutic Interventions

The patient was prescribed optical correction for the presbyopic complaints and referred for follow-up of the fundus finding every 6 months. The need for active surveillance was emphasized due to the potential risk of malignant transformation of the lesion.

## 3. Discussion

In clinical practice, the presence of pigmented tumor growth in the fundus is the most significant sign of malignancy. Most choroidal nevi remain stable for years, unlike malignant melanoma, which has a tendency for rapid growth. Another patient of ours, who had a small pigmented lesion in the fundus (4.8 mm at the base, with a prominence of 1.82 mm), did not appear for control examinations and for 4 years showed a significant increase in size: 16.32 mm at the base and a prominence of 8.49 mm. In the same patient, after enucleation, the histological diagnosis confirmed malignant melanoma. It is considered that a growth rate of nevi exceeding 3% of the initial size per year raises suspicion of malignancy, while an increase of up to 1% within 5 years suggests the lesion is at no risk of transforming into melanoma [12]. In our clinical case, no dynamics in the size of the tumor formation were detected within one year.

The signs that must be observed in order to assess the risk of malignant transformation of nevi are grouped into the acronym TFSOMUHHD (“to find small ocular melanoma using helpful hints daily”), which includes [13]

1. T—thickness—tumor thickness (prominence) greater than 2 mm, determined by ultrasound examination, and lesion diameter greater than 5 mm;

2. F—fluid—presence of subretinal fluid on optical coherence tomography;

3. S—symptoms—decreased vision, photopsia, floaters;

4. O—orange—orange lipofuscin pigment of the tumor and presence of autofluorescence upon examination of the fundus;

5. M—margin—the edge of the lesion must be less than 3 mm from the optic disc;

6. UH—ultrasound hollowness—an ultrasound sign, when the base of the tumor appears less echogenic;

7. H—halo—lack of halo during ophthalmoscopy;

8. D—drusen—absence of drusen on ophthalmoscopy.

According to Chien and colleagues, the presence of three or more of these risk factors implies a greater than 50% chance of transformation into melanoma within 5 years [13]. Shields et al. determined the 5-year risk of tumor growth at 4% in the absence of risk factors, 45% when two risk factors are present, and 56% if all risk factors are present [14].

In 10% of all patients with choroidal nevus, subjective complaints are reported. These are usually cases with nevi located subfoveally or juxtapapillarily. Reduced visual acuity is also observed with macular edema and subretinal fluid [15]. Some studies have reported visual field defects in eyes with choroidal melanoma [16,17,18]. The subretinal fluid in a choroidal nevus may be due to leakage through the retinal pigment epithelium (RPE) and, less commonly, due to a choroidal neovascular membrane. These changes are well visualized on FA and OCT. The ophthalmoscopic evaluation of benign choroidal lesions is crucial when determining risk factors for malignancy. The presence of orange pigment on the surface of the tumor, the absence of drusen, and the proximity of the tumor to the papilla of the optic nerve are signs of higher risk [10]. Drusen and pigment epithelial atrophy indicate a chronic and stable process, which is why they are considered a sign of benignity.

Stoyukhina classified nevi according to OCT findings into nevi without risk and nevi with risk of transformation into melanoma [12]. The author found that the increase in size of the choroidal nevi in the choroid leads to dystrophic processes and changes in the RPE. The impaired pumping function of the RPE is associated with impaired trophism and development of atrophic changes in the retina adjacent to the nevus, which is considered a benign characteristic. Nevi that are imaged on OCT with neuroepithelial detachment and the presence of subretinal fluid are considered a risk factor for transition to malignant transformation. In our patient, no such signs were found on OCT.

Ultrasound B-scan is essential when documenting and monitoring the growth of intraocular lesions over time. Most choroidal nevi are flat and cannot be detected by ultrasound. A-ultrasound can differentiate the internal structure of the tumor in a differential melanoma diagnosis, which shows low to medium internal ultrasound reflectivity with posterior attenuation, unlike the pigmented nevus, which is characterized by high internal reflectivity out of posterior attenuation (kappa angle = 0). In our patient, the ultrasound revealed a pigmented nevus smaller in size than the diameter measured on the native photographs. The small prominence (0.57 mm), measured by ultrasound, which showed no signs of growth within a year, is considered a benign lesion.

The differential diagnosis of choroidal nevi is mainly made with malignant melanoma, with benign congenital hypertrophy of the pigment epithelium (BCHPE) and rarely with melanocytoma. The suspicious choroidal neoplasms are characterized by a larger size, the presence of visual symptoms, serous retinal detachment, and the presence of orange pigment, and on FA—points of leakage from the tumor’s own vascular network, the typical ultrasonic characteristics described above, and potential for growth during subsequent examinations. Choroidal nevi are more echogenic than B-echography melanomas and show no signs of choroidal excavation, which is observed in melanomas. The benign congenital hypertrophy of the pigment epithelium is a flat, highly pigmented lesion with polycyclic outlines that has a slowly progressive diameter but remains flat. It is often much larger and more heavily pigmented than the nevus. In fluorescein angiogram lesions, blackened hypo-fluorescence is noted in all phases. Ultrasonography typically shows that BCHPE is flat to minimally elevated and slightly hyper-reflective. Melanocytoma is usually located at the level of the optic disc nerve; it is a slightly prominent round benign tumor—so pigmented that it appears black. Melanocytomas are most often asymptomatic, but small defects in the visual field due to compression of the optic fibers are possible in larger tumors [19]. The ultrasound examination in melanocytoma detects an elevated lesion in the optic disk nerve, which is characterized by high and regular internal reflectivity.

The prognosis for patients with choroidal nevus depends on the risk of malignant transformation and, accordingly, the likelihood of melanomas forming metastases. Since this risk is directly related to their size, it is important to diagnose and treat them early. For instance, Diener et al. found that a malignant melanoma thickness of less than 3 mm leads to 16% metastases, between 3 and 8 mm—32% metastases, and a tumor greater than 8 mm—53% [20]. In a study of 1329 small pigmented choroidal tumors, Shields established that the 5-year risk of metastases for tumors less than 3 mm was only 3% [21].

The possible treatment of a suspected choroidal nevus without documented growth must be considered on a case-by-case basis, depending on the number of risk factors, as well as the location of the tumor, due to the risk to visual function after treatment—especially in lesions located in the macula or near the optic nerve [22].

## 4. Conclusions

This case of a giant pigmented nevus of the choroid highlights the importance of correct diagnosis and differentiation from malignant melanoma, especially in young patients, due to the different therapeutic approach and different prognosis. Despite the diverse methods for documenting and monitoring choroidal pigmented nevi, the differential diagnosis with regard to small uveal melanomas remains difficult. In all cases of choroidal nevi, especially with larger sizes, frequent check-ups are recommended to exclude growth and change in the type of lesion. Monitoring patients allows for the early detection of signs of malignant transformation and timely treatment, which can preserve patients’ lives.

## Data Availability

The original data presented in this study are available on reasonable request from the corresponding author. The data are not publicly available due to privacy concerns.
